# Comparison of High-Flow Nasal Cannula Versus Conventional Oxygen Therapy After Extubation in Children Undergoing Cardiac Surgery: A Meta-analysis

**DOI:** 10.7759/cureus.36922

**Published:** 2023-03-30

**Authors:** Jithin Karedath, Modather I Hatamleh, Rushna Haseeb, Rameeza Stephana Cela, Syed Asjad Tauheed Zaidi, Sandipkumar S Chaudhari, Zainab Naseer, Neelum Ali

**Affiliations:** 1 Internal Medicine, King's College Hospital NHS Foundation Trust, London, GBR; 2 Internal Medicine, King Abdullah University Hospital, Amman, JOR; 3 Internal Medicine, Jinnah Hospital Lahore/Allama Iqbal Medical College, Lahore, PAK; 4 Medicine, American Institute of Integrative Sciences, New York, USA; 5 General Practice, Shalamar Medical and Dental College, Lahore, PAK; 6 General Practice, Lions General Hospital, Mehsana, IND; 7 Internal Medicine, American Institute of Integrative Sciences, New York, USA; 8 Internal Medicine, University of Health Sciences, Lahore, PAK

**Keywords:** meta-analysis, cardiac surgery, children, high flow nasal cannula, conventional oxygen therapy

## Abstract

This meta-analysis aims to compare high-flow nasal cannula (HFNC) and conventional oxygen therapy (COT) post-extubation in pediatric cardiac surgical patients. The present meta-analysis was conducted according to the Preferred Reporting Items for Systematic Reviews and Meta-Analyses (PRISMA) guidelines. Two authors independently searched three electronic databases including PubMed, Embase, and the Cochrane Library to identify relevant articles published in English from inception to February 2023. Searching was conducted using keywords and medical subject headings (MeSH), which included "conventional oxygen therapy," "high-flow nasal cannula," "extubation," "pediatrics," and "cardiac surgery."

Our primary outcome was extubation failure defined as the need for reintubation within 24 to 72 hours after planned extubation. Secondary outcomes assessed in this meta-analysis included partial pressure of arterial oxygen (PaO2), partial pressure of arterial carbon dioxide (PaCO2), and the ratio of PaO2 and FiO2 (fraction of inspired oxygen). A total of three studies were included in the meta-analysis, with a total of 227 patients.

No significant difference was found between the two groups (the HFNC group and the COT group) in terms of reintubation (RR: 0.88, 95% CI: 0.34, 2.30, p-value: 0.80). Pooled meta-analysis showed that PaO2 was significantly greater in patients receiving HFNC at six hours (MD: 33.73, 95% CI: 18.33, 49.14, p-value<0.001), at 12 hours (MD: 44.90, 95% CI: 28.59, 61.22, p-value<0.001) and at 24 hours (MD: 43.53, 95% CI: 29.16, 57.91, p-value<0.001) of extubation. PaCO2 was significantly lower in patients receiving HFNC at six hours (MD: -5.40, 95% CI: -7.94, -2.85, p-value<0.001) and at 12 hours (MD: -5.93, 95% CI: -9.78, -2.09, p-value<0.001) of extubation. However, no significant difference was reported between the two groups after 24 hours of extubation (MD: -0.84, 95% CI: -9.04, 7.37, p-value: 0.84) and PaO2/FiO2 was significantly greater in patients receiving HFNC at six hours (MD: 64.14, 95% CI: 36.10, 92.17, p-value<0.001), at 12 hours (MD: 70.73, 95% CI: 20.46, 121.01, p-value<0.001) and at 24 hours (MD: 82.18, 95% CI: 50.03, 114.32, p-value<0.001) of intubation.

In conclusion, the meta-analysis revealed that compared with COT, HFNC significantly increased PaO2 and the ratio of PaO2 to FiO2, and decreased PaCO2. No significant differences were observed in the rate of reintubation between the two groups. This is the first meta-analysis comparing HFNC and COT in pediatric cardiac surgical patients

## Introduction and background

Mechanical ventilation is associated with significant complications that are influenced by time. Longer intubation duration results in a higher occurrence of complications such as ventilator-associated pneumonia (VAP) and increased mortality [1]. Removing the endotracheal tube (extubation) is beneficial as it reduces the chances of VAP, removes the breathing workload imposed by the endotracheal tube, and enhances patient comfort [2]. Nonetheless, following the removal of the endotracheal tube, the functional residual capacity that was maintained by positive end-expiratory pressure (PEEP) during invasive ventilation may decline abruptly, resulting in hypoxemia and extubation failure [3]. Extubation failure is associated with an increase in both the length of intensive care unit (ICU) stay and the rate of mortality [4]. Hence, adequate respiratory support is important to prevent the need for reintubation.

Conventional oxygen therapy (COT) and high-flow nasal cannula (HFNC) are two common methods of providing adequate respiratory support after extubation. HFNC ventilation is a method of providing a combination of air and oxygen to a patient using a moistened circuit at extremely high flow rates that go beyond the patient's natural breathing rate [5]. The technique provides heated and moistened gases and some degree of continuous positive airway pressure (CPAP), but the amount of pressure is not always easy to predict [6]. The use of HFNC has been proven to reduce airway resistance and to flush nasopharyngeal dead space, leading to decreased work of breathing, which favors the elimination of carbon dioxide and bronchial secretions [7]. COT is also used as the main supportive treatment given to patients after planned extubation and can be delivered through masks or nasal prongs. However, the maximum oxygen flow rates that these devices can deliver are limited [8]. COT is usually delivered by a simple face mask or nasal cannula with the same target SpO2. In the pediatric population, oxygen is delivered with a flow meter from wall oxygen and humidification with a closed sterile water system at room temperature [9].

COT involves the delivery of oxygen through a nasal cannula, face mask, or other similar devices, typically at a flow rate of up to 15 liters per minute (LPM). The amount of oxygen delivered is dependent on the patient's respiratory rate and tidal volume, as well as the device used [6]. On the other hand, HFNC is a form of respiratory support that delivers humidified and heated oxygen at high flow rates of up to 60 LPM. HFNC devices are designed to deliver a precise mixture of air and oxygen, which can be adjusted according to the patient's needs [9].

Factors such as surgical incision, general anesthesia, the intensity of surgical manipulation, the number of drains, and cardiopulmonary bypass (CPB) may incline patients to changes in pulmonary functions that are highly related to the onset of respiratory complications post-cardiac surgery [10]. The most frequent post-surgical complications observed in children undergoing cardiac surgery include respiratory stridor caused by swelling of the glottis, atelectasis, pulmonary edema, pleural effusion, chylothorax, diaphragmatic dysfunction, and pneumonia resulting from mechanical ventilation. These and other complications can potentially result in respiratory failure [11]. Indeed, managing the period after a patient has had their breathing tube removed can be complicated, particularly in this group of patients. If traditional oxygen therapy proves inadequate, the application of continuous positive airway pressure (CPAP) or non-invasive ventilation (NIV) may be required [12].

Despite the increased use of HFNC in the pediatric ICU, the clinical and physiological effect of HFNC therapy on the pediatric population with respiratory distress after cardiac surgery has not been thoroughly investigated [13]. Therefore, the present meta-analysis was conducted to compare the effect of HFNC and COT using a larger sample size. The aim of this meta-analysis was to compare HFNC and COT post-extubation in pediatric cardiac surgical patients.

## Review

Methodology

The present meta-analysis was conducted based on the Preferred Reporting Items for Systematic Reviews and Meta-Analyses (PRISMA) guidelines.

Data Sources and Search Strategy

Two authors independently searched three electronic databases including PubMed, Embase, and the Cochrane Library to identify relevant articles published in the English language from inception to February 2023. Searching was conducted using keywords and medical subject headings (MeSH), which included "Conventional oxygen therapy," "High-flow nasal cannula," "extubation," "pediatrics," and "cardiac surgery." All eligible records were imported into EndNote version X9 (Clarivate, Philadelphia, PA). After removing duplicates, two authors independently screened the titles and abstracts of studies. The full-text of eligible records was retrieved, and detailed screening was conducted based on pre-defined inclusion and exclusion criteria. Any disagreement in the process of searching and study selection was resolved via discussion or consensus of the principal investigator.

Eligibility Criteria

The inclusion criteria for the included studies were as follows: (1) study design: cohort (prospective and retrospective studies) and randomized control trials (RCT); (2) participants: children under 18 years following extubation after pediatric cardiac surgery; (3) intervention: comparison of COT and HFNC; (4) outcomes: post-extubation failure, partial pressure of arterial oxygen (PaO2), and partial pressure of arterial carbon dioxide (PaCO2). The exclusion criteria were case reports, cross-over studies, case series, animal studies, cross-sectional studies, or patients older than 18 years.

Data Extraction

Two investigators independently screened and extracted the studies based on the eligibility criteria. The information extracted from the eligible studies included the name of the first author, year of publication, study design, sample size, study population, interventional details, and outcomes. Extracted data were entered into RevMan software for data analysis.

The quality of the included studies was independently assessed by two authors using Cochrane Collaboration’s tool for RCTs and the Newcastle-Ottawa quality assessment form for cohort studies. Any disagreement in the process of data extraction and study quality assessment was resolved via discussion or consensus with the third author if needed.

Outcome Measures

Our primary outcome was extubation failure defined as the need for reintubation within 24 to 72 hours after a planned extubation. Secondary outcomes assessed in this meta-analysis included partial pressure of arterial oxygen (PaO2), partial pressure of arterial carbon dioxide (PaCO2), and ratio of PaO2 and FiO2 (fraction of inspired oxygen).

Data Analysis

We used RevMan 5.4.1 (Cochrane IMS, Oxford, United Kingdom) for all analyses. For dichotomous outcomes, results were reported using risk ratio (RR) with their 95% confidence interval (CI). For continuous outcomes, results were reported using mean differences (MD) with 95% CI. Heterogeneity among the study results was reported using I-square statistics. In case of an I-square value of >50%, a random-effects model was used. Otherwise, we used the fixed effect model to compute effect measures.

Results

**Figure 1:**
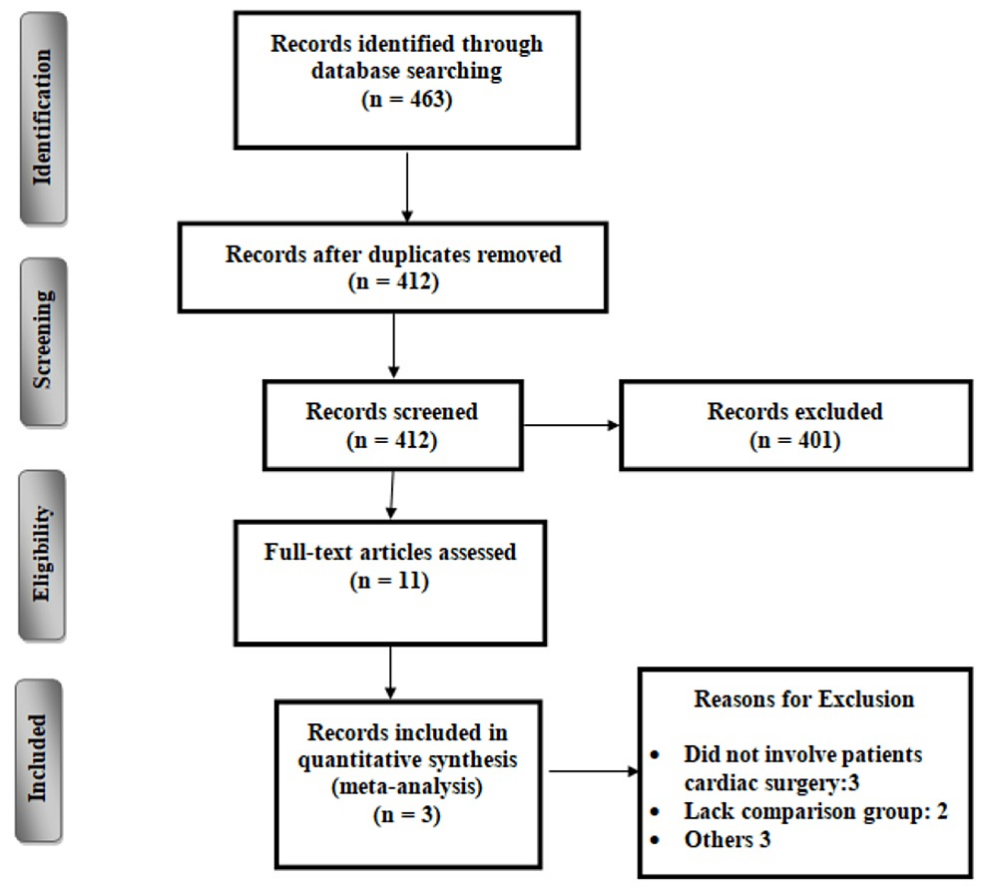
PRISMA flowchart of selection of studies

Initially, 463 articles were obtained from the aforementioned electronic databases. After removing duplicates, 412 articles were included in the initial level screening. Of them, 11 studies were included in full-text review. Finally, three studies were included in the meta-analysis, with a total of 227 patients. The PRISMA flowchart of the selection of studies is shown in Figure 1. The characteristics of included studies are shown in Table 1. Out of three studies, two were RCTs and 1 was retrospective. No significant differences were found between the two groups in any of the included studies between two groups in terms of mechanical ventilation, cyanosis, and cardiopulmonary bypass (CPB) time. Quality assessment is shown in Table 2.

**Table 1:** Characteristics of included studies RCT: randomized control trial; CPB: cardiopulmonary bypass; HFNC: high-flow nasal cannula; COT: conventional oxygen therapy

Author Name	Year	Study Design	Groups	COT Mode	Sample Size	Mechanical Ventilation (Hours)	CPB Time (Minutes)	Cyanosis (n)
Burra et al. [14]	2019	RCT	HFNC		25	23.6 vs 23.2	72.5 vs 79.1	8 vs 7
			COT	Face Mask	25			
Şavluk et al. [12]	2021	Retrospective	HFNC		45	17.6 vs 17.1	134.6 vs 130.4	13 vs 10
			COT	Face Mask	45			
Testa et al. [15]	2014	RCT	HFNC		43	24 vs 24	155 vs 157	14 vs 10
			COT	Face Mask	44			

**Table 2:** Quality assessment of included studies

Quality assessment for randomized control trial					
Study ID	Selection Bias	Performance	Attrition	Reporting	Other
Burra et al. [14]	Yes	No	Yes	No	No
Testa et al. [15]	Yes	No	No	No	No
Quality Assessment for Retrospective Studies					
Study ID	Selection	Comparability	Outcome	Overall	
Şavluk et al. [12]	2	1	2	Fair	

Meta-analysis of outcomes

Reintubation

**Figure 2:**
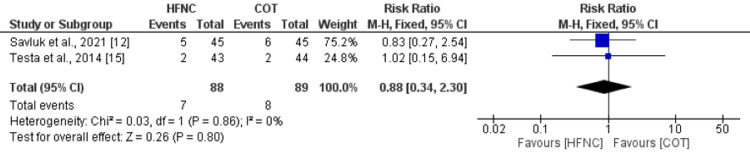
Forest plot of comparison of reintubation between HFNC and COT HFNC: high-flow nasal cannula; COT: conventional oxygen therapy Sources: Şavluk et al., Testa et al. [12, 15]

Two of three studies compared the incidence of reintubation between HFNC and COT groups. A total of 15 out of 177 patients were reintubated. No significant difference was found between the two groups in terms of reintubation (RR: 0.88, 95% CI: 0.34, 2.30, p-value: 0.80) as shown in Figure 2. No heterogeneity was found among the study results (I-square: 0%).

Partial Pressure of Arterial Oxygen (PaO2)

All three studies assessed PaO2 at six, 12, and 24 hours of extubation. Pooled meta-analysis showed that PaO2 was significantly greater in patients receiving HFNC at six hours (MD: 33.73, 95% CI: 18.33, 49.14, p-value<0.001), at 12 hours (MD: 44.90, 95% CI: 28.59, 61.22, p-value<0.001) and at 24 hours (MD: 43.53, 95% CI: 29.16, 57.91, p-value<0.001) of intubation. No heterogeneity was reported among the study results as shown in Table 3.

**Table 3:** Comparison of PaO2, PaCO2, and PaO2/FiO2 at six hours, 12 hours, and 24 hours between the two groups PaO2: partial pressure of arterial oxygen; PaCO2: partial pressure of arterial carbon dioxide; FiO2: fraction of inspired oxygen * Significant at p-value<0.05

Outcome	Time	MD (95% CI)	I-square
Pa02	6 hours	33.73 (18.33, 49.14)*	0%
	12 hours	44.90 (28.59, 61.22)*	0%
	24 hours	43.53 (29.16, 57.91)*	0%
PaCO2	6 hours	-5.40 (-7.94, -2.85)*	0%
	12 hours	-5.93 (-9.78, -2.09)*	52%
	24 hours	-0.84 (-9.04, 7.37)	92%
Ratio of PaO2 to FiO2	6 hours	64.14 (36.10, 92.17)*	24%
	12 hours	70.73 (20.46, 121.01)*	57%
	24 hours	82.18 (50.03, 114.32)*	44%

Partial Pressure of Arterial Carbon Dioxide (PaCO2)

All three studies assessed PaCO2 at six, 12, and 24 hours of extubation. Pooled meta-analysis showed that PaCO2 was significantly lower in patients receiving HFNC at six hours (MD: -5.40, 95% CI: -7.94, -2.85, p-value<0.001) and at 12 hours (MD: -5.93, 95% CI: -9.78, -2.09, p-value<0.001) of extubation. However, no significant difference was reported between the two groups after 24 hours of extubation (MD: -0.84, 95% CI: -9.04, 7.37, p-value: 0.84) as shown in Table 3.

Ratio of PaO2 to FiO2

All three studies assessed PaO2/FiO2 at six, 12, and 24 hours of extubation. Pooled meta-analysis showed that PaO2/FiO2 was significantly greater in patients receiving HFNC at six hours (MD: 64.14, 95% CI: 36.10, 92.17, p-value<0.001), at 12 hours (MD: 70.73, 95% CI: 20.46, 121.01, p-value<0.001) and at 24 hours (MD: 82.18, 95% CI: 50.03, 114.32, p-value<0.001) of intubation. No heterogeneity was reported among the study results as shown in Table 3.

Adverse Events

Burra et al. and Şavluk et al. [12, 14] did not report any complications in any of the two groups. However, Testa et al. [15] found one case of chylothorax and one case of pneumothorax in each group; two and three cases of pleural effusions were reported in COT and HFNC groups respectively. The rate of atelectasis was also equal in the two groups [15].

Discussion

The present meta-analysis, which includes three studies with a total of 227 patients (HFNC group: 113 patients; COT group: 114 patients), showed that compared with COT, HFNC significantly increases PaO2 and the ratio of PaO2 to FiO2, and decreases PaCO2. No significant differences were reported in the reintubation rate. To the best of our knowledge, this is the first meta-analysis comparing HFNC and COT post-extubation in pediatric cardiac surgical patients. Previous human and animal studies have shown that HFNC can deliver more adequate inspiratory flow, humidify and warm the airways, and flush the nasopharyngeal dead space, creating a positive airway pressure, decreasing the work of breathing and respiratory rate, improving dyspnea and oxygenation, and enhancing comfort [16-17].

A study conducted by Roca et al. found a significant improvement in arterial blood gases and respiratory parameters in adults as early as 30 minutes of HFNC compared to 30 minutes of COT [8]. Our meta-analysis showed a beneficial impact of HFNC in improving PaO2 levels at six, 12, and 24 hours post-extubation. The patients who received HFNC also had better CO2 elimination compared to the patients receiving COT, which was statistically significant at six and 12 hours post-extubation. However, at 24 hours of extubation, PaCO2 was not significantly different between the two groups. The PaO2/FiO2 ratio variation was also significant between the two groups at six, 12, and 24 hours following extubation, with higher values in the HFNC group.

Woodhead et al. conducted a study on premature infants using a randomized crossover design. They compared the use of HFNC at a flow rate of 3.1 ± 0.6 L/min with non-humidified high-flow oxygen at a flow rate of 1.8 ± 0.4 L/min. The study showed that the HFNC group had a significantly lower reintubation rate [18]. In another study, Holleman-Duray et al. conducted a retrospective analysis of 114 premature infants. They compared the use of HFNC at a flow rate of 4-6 L/min with ventilator CPAP at a pressure of 8 cm H2O. The study concluded that premature infants who were extubated to HFNC spent significantly fewer days on the ventilator [19].

Moreover, in our study, 15 patients were intubated after extubation. Seven patients were in the HFNC group and eight patients were in the COT group. In the present meta-analysis, no significant difference was found between the two groups regarding the incidence of reintubation. However, in another study comparing the impacts of HFNC therapy and the venturi mask, it was demonstrated that fewer reintubations were required in the HFNC group [20].

This study has also found that HFNC is a safe practice in the pediatric population. No complications such as gastric distension or nasal ulcer were reported in any of the studies. A study conducted by Campbell et al. found no differences in the reintubation rate and incidence of complications between the HFNC and CPAP groups [21]. HFNC is usually considered safe for use in the general pediatric ward, emergency department, and pediatric intensive care unit. However, one well-known risk associated with HFNC is barotrauma, which can lead to conditions such as air-trapping, pneumothorax, and pneumomediastinum [22]. In one of the studies included in the present meta-analysis, one case of pneumothorax was reported in each of the two groups [15].

Study Limitations

The present study had several limitations. Firstly, only three studies were included, which resulted in a relatively small pooled sample size of 227. This limited the generalizability of the findings to a broader population. Secondly, due to the limited data availability, we were unable to perform subgroup analysis, which could have provided additional insights into the factors that may influence the outcomes of interest. Finally, we did not perform a publication bias analysis because of the limited number of studies included in the meta-analysis. Therefore, caution should be exercised when interpreting the results of this study, and further research is warranted to validate these findings.

## Conclusions

In conclusion, this meta-analysis examined the effects of high-flow nasal cannula (HFNC) versus conventional oxygen therapy (COT) post-extubation in pediatric cardiac surgical patients. Three studies with a total of 227 patients were included in the analysis, with 113 patients in the HFNC group and 114 patients in the COT group. The meta-analysis revealed that compared with COT, HFNC significantly increased the partial pressure of arterial oxygen (PaO2) and the ratio of PaO2 to FiO2, and decreased the partial pressure of arterial carbon dioxide (PaCO2). No significant differences were observed in the rate of reintubation between the two groups. This is the first meta-analysis comparing HFNC and COT in pediatric cardiac surgical patients. Overall, HFNC showed beneficial effects on oxygenation and ventilation parameters compared to COT, which may be important in the post-extubation period of these patients. The use of HFNC in pediatric cardiac surgical patients can be considered a safe option as well to decrease the need for noninvasive post-extubation respiratory support. Further studies with larger sample sizes are warranted to validate these findings.
